# Genomic Breakpoint Characterization and Transcriptome Analysis of Metastatic, Recurrent Desmoplastic Small Round Cell Tumor

**DOI:** 10.1155/2023/6686702

**Published:** 2023-07-06

**Authors:** Justin W. Magrath, Dane A. Flinchum, Alifiani B. Hartono, Ilon N. Goldberg, Madelyn Espinosa-Cotton, Krzysztof Moroz, Nai-Kong V. Cheung, Sean B. Lee

**Affiliations:** ^1^Department of Pathology and Laboratory Medicine, Tulane University School of Medicine, 1430 Tulane Ave. New Orleans, LA, USA; ^2^Department of Pediatrics, Memorial Sloan Kettering Cancer Center, New York, NY, USA

## Abstract

Desmoplastic small round cell tumor (DSRCT) is a rare pediatric cancer caused by the *EWSR1-WT1* fusion oncogene. Despite initial response to chemotherapy, DSRCT has a recurrence rate of over 80% leading to poor patient prognosis with a 5-year survival rate of only 15–25%. Owing to the rarity of DSRCT, sample scarcity is a barrier in understanding DSRCT biology and developing effective therapies. Utilizing a novel pair of primary and recurrent DSRCTs, we present the first map of DSRCT genomic breakpoints and the first comparison of gene expression alterations between primary and recurrent DSRCT. Our genomic breakpoint map includes the lone previously published DSRCT genomic breakpoint, the breakpoint from our novel primary/recurrent DSRCT pair, as well as the breakpoints of five available DSRCT cell lines and five additional DSRCTs. All mapped breakpoints were unique and most breakpoints included a 1–3 base pair microhomology suggesting microhomology-mediated end-joining as the mechanism of translocation fusion and providing novel insights into the etiology of DSRCT. Through RNA-sequencing analysis, we identified altered genes and pathways between primary and recurrent DSRCTs. Upregulated pathways in the recurrent tumor included several DNA repair and mRNA splicing-related pathways, while downregulated pathways included immune system function and focal adhesion. We further found higher expression of the *EWSR1-WT1* upregulated gene set in the recurrent tumor as compared to the primary tumor and lower expression of the *EWSR1-WT1* downregulated gene set, suggesting the *EWSR1-WT1* fusion continues to play a prominent role in recurrent tumors. The identified pathways including upregulation of DNA repair and downregulation of immune system function may help explain DSRCT's high rate of recurrence and can be utilized to improve the understanding of DSRCT biology and identify novel therapies to both help prevent recurrence and treat recurrent tumors.

## 1. Introduction

Desmoplastic small round cell tumor (DSRCT) is a rare and aggressive pediatric cancer that most commonly presents in the abdominal or pelvic region [1–3]. DSRCT is caused by a translocation between the Ewing sarcoma breakpoint region 1 (*EWSR1*) gene on chromosome 22 and the Wilms tumor 1 (*WT1*) gene on chromosome 11, leading to the establishment of the *EWSR1-WT1* fusion oncogene which alters gene expression and leads to malignancy [4–7]. While DSRCT is susceptible to chemotherapy which can facilitate tumor shrinkage, recurrence is frequent and the DSRCT survival rate is only 15–25% [2, 3, 8, 9]. DSRCT is also notable for its extremely high level of metastasis. At the time of diagnosis, 90% of DSRCT patients have intraperitoneal metastases and 25–43% of cases have extraperitoneal metastases, contributing to the lethality of this tumor [2, 3].

Due to the rarity of DSRCT, estimated at fewer than 50 cases per year in the United States, sample procurement has been a limiting factor to scientific discovery [10–12]. While for a long time there was only one commonly available DSRCT cell line (JN-DSRCT-1), over the past several years additional DSRCT cell lines have been established [13, 14]. The first papers utilizing RNA-sequencing (RNA-seq) both on cell lines and tumors have also been published recently, providing much needed data to advance the study of this extremely aggressive malignancy [7, 15, 16]. However, despite this recent influx of DSRCT samples and resources, many fundamental questions remain unanswered. The DSRCT cell of origin is unknown and even the pattern of genomic breakpoints that leads to the formation of the critical *EWSR1-WT1* fusion gene remains uninvestigated [15]. While RT-PCR was used to identify the adjoining exons in DSRCT cell lines and integrated mutation profiling of actionable cancer targets (MSK-IMPACT) exome sequencing identified the adjoining exons in 17 DSRCTs, only one study examining one tumor has identified a specific DSRCT genomic breakpoint [13, 17, 18]. In comparison, genomic breakpoints have been identified for over 50 cases of chronic myeloid leukemia [19–21], 49 cases of Ewing sarcoma [22], 12 cases of synovial sarcoma [23, 24], and 3 cases of alveolar rhabdomyosarcoma [25], establishing patterns that provide insight into the mechanism of original tumor formation including whether the process likely employed nonhomologous end-joining (NHEJ), homologous recombination (HR), or microhomology-mediated end-joining (MMEJ).

Another area that remains completely uninvestigated is the difference between primary and recurrent and/or metastatic DSRCTs. In fact, no study to date has examined the differences between primary and recurrent DSRCTs. Given DSRCT's initial propensity to respond to chemotherapy but eventual resistance and recurrence, understanding the mechanisms DSRCT employs to evade both chemotherapeutics and the immune system will be critical in devising effective DSRCT treatments.

In this study, we utilize a novel pair of primary and recurrent DSRCTs derived from the same patient five years apart to investigate these outstanding questions within the DSRCT field. After confirming the presence of the DSRCT fusion gene in both the primary and recurrent tumor, we utilize a novel PCR strategy to identify the specific genomic breakpoints of our new patient tumor pair as well as the genomic breakpoints of the five available DSRCT cell lines and five additional DSRCTs. Using these data and the previously published DSRCT genomic breakpoint case study, we establish the first-in-kind map of DSRCT genomic breakpoints which suggests the role for MMEJ in the formation of the *EWSR1-WT1* fusion gene. We further perform RNA sequencing on the first analyzed primary-recurrent DSRCT pair and identify pathways likely to play a role in chemotherapy evasion, tumor recurrence, and metastasis including increases in homologous recombination, alternation in RNA splicing, and reduction in cytokine secretion. The RNA sequencing analysis also for the first time analyzes the role of the EWSR1-WT1 signature in recurrent/metastatic DSRCTs and the potential susceptibility of these tumors to fusion gene targeting treatments.

## 2. Materials and Methods

### 2.1. Tumor Samples

Frozen samples of primary (T2911) and recurrent (T2912) tumors were received from the Louisiana Cancer Research Center Biospecimen Core (IRB Project 0M600). Frozen and OCT-embedded primary tumors MSK4832, MSK4991, MSK5070, MSK5117, and MSK5338 were received from Memorial Sloan Kettering Cancer Center approved under IRB/Privacy Board 21-282. Patients provided their written and informed consent. Sample information is detailed in Supplementary Table 1.

### 2.2. Cell Lines

JN-DSRCT-1, BER-DSRCT, BOD-DSRCT, SK-DSRCT1, and SK-DSRCT2 cell lines have been previously described [13, 26, 27] and validated to harbor the defining *EWSR1-WT1* fusion. Cells were grown in DMEM/F12 media supplemented with 10% FBS, 2 mM L-glutamine, 100 U/mL penicillin, and 100 mg/mL streptomycin (Thermo Fisher, Waltham, MA).

### 2.3. Transcript Junction Identification

Total RNA was isolated with RNA-STAT60 (Tel-Test, Friendswood, TX). 500 ng of RNA was reverse-transcribed to form cDNA using iScript cDNA synthesis kit (Bio-Rad, Hercules, CA). Primers derived from exon 7 of *EWSR1* and exon 10 of *WT1* were used to amplify PCR products spanning the fusion gene. PCR amplicons were run on a 2% agarose gel for size comparison and rapid transcript breakpoint identification. All PCR amplicons were sequenced, and BER-DSRCT, SK-DSRCT2, and JN-DSRCT-1, whose RNA junctions have been previously determined, were used as controls. Primers are listed in Supplementary Table 2.

### 2.4. Genomic Breakpoint Identification

Genomic DNA was isolated from frozen primary DSRCT specimens with DNazol (Thermo Fisher) per the manufacturer's protocol or with a standard extraction buffer (10 mM NaCl, 20 mM Tris-HCl, pH 8.0, 1 mM EDTA, and 0.5% SDS) followed by addition of proteinase K digestion overnight at 55°C, phenol/chloroform extraction, and ethanol precipitation. In some cases, DNA was further purified using miniprep DNA columns (Qiagen) before PCR amplification. Two sets of forward PCR primers derived from *EWSR1* intron 7 or 9 were used for each genomic DNA PCR based on the identified transcript junctions from the RT-PCR analysis. A common set of three reverse primers in intron 7 of *WT1* was used. PCR products were analyzed on 1% agarose gel and purified using DNA Clean and Concentrator kit (Zymo Research, Irvine, CA). 70 ng of PCR product was used for Sanger sequencing with the appropriate forward primer to identify the exact genomic breakpoint. Primers are listed in Supplementary Table 2.

### 2.5. RNA Sequencing and Analysis

For RNA-seq analysis, total RNAs were prepared using RNeasy Plus kit (Qiagen, Hilden, Germany). Sequencing libraries were constructed from 500 ng of total RNA using the Illumina TruSeq Stranded Total RNA kit with ribo zero (San Diego, CA) following the manufacturer's instruction. The fragment size of RNAseq libraries was verified using the Agilent 2100 Bioanalyzer (Agilent, Santa Clara, CA), and the concentrations were determined using Qubit instrument (Thermo Fisher). RNA-seq libraries were loaded onto Illumina NovaSeq 6000 (San Diego, CA) for 75 bp paired-end read sequencing. Fastq files were generated using bcl2fastq software [28]. Gene counts were generated by pseudoalignment to the human ENSEMBL transcriptome version 109 using kallisto [29]. Only genes with TPM >0.5 for both samples were included for further analysis. RNAseq data have been submitted to GEO (GSE230603). Normal tissue RNA sequencing samples from TCGA-LIHC (TCGA-DD-A1EL, TCGA-DD-A39X, TCGA-BC-A216, TCGA-FV-A2QR, and TCGA-BD-A3EP) and TCGA-SARC (TCGA-FX-A2QS and TCGA-K1-A3PO) were obtained using the Genomic Data Commons portal. Differential gene expression analysis was performed with DESeq2 [30]. Gene Set Enrichment Analysis was performed using the clusterProfiler package in Bioconductor [31, 32].

### 2.6. Real-Time qPCR Analysis

RNAs from cells and primary tumors were used to generate cDNAs as described above. Relative transcript levels were analyzed by real-time qPCR using SYBR Green (SsoAdvanced Universal SYBR Green Supermix, Bio-Rad) and calculated by the comparative Ct method normalized against human *β*-actin. Primers are listed in Supplementary Table 3.

## 3. Results

### 3.1. Identification of a Case of Primary and Metastatic DSRCT

**Figure 1:**
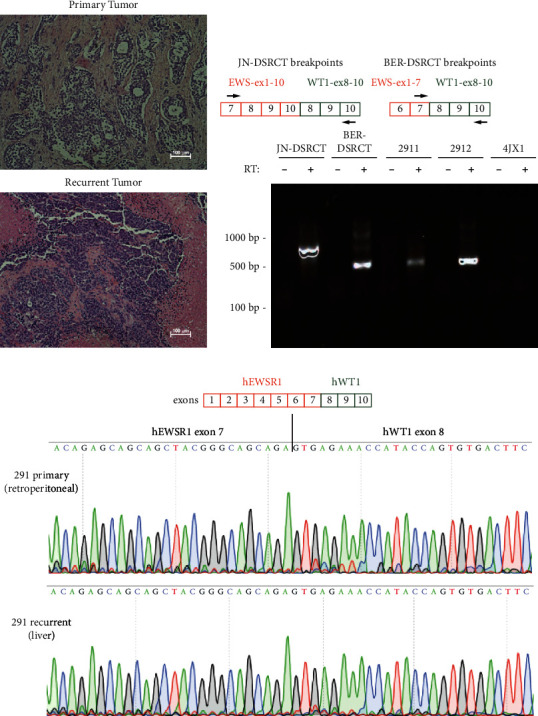
DSRCT validation: (a) H&E staining of primary and recurrent DSRCTs from the same patient (scale bar = 100 *µ*m). (b) PCR amplification of the *EWSR-WT1* fusion gene from cDNA derived from JN-DSRCT-1 and BER-DSRCT positive controls, the primary tumor (2911) and recurrent tumor (2912), or the 4JX1 negative control. (c) Histograms of Sanger sequencing from primary and recurrent DSRCTs show seamless transition between *EWSR1* exon 7 and *WT1* exon 8.

Samples of primary and metastatic DSRCT from the lone DSRCT patient treated in New Orleans in recent years were obtained from the Louisiana Cancer Research Center Biospecimen Core. DSRCT samples were obtained from surgical resection of retroperitoneal pelvic mass when the patient was 24 years of age. At that time, liver capsular implants were present and removed, but the liver parenchyma was not directly involved. Five years later, the patient developed parenchymal liver metastases that were sampled and confirmed to represent metastatic DSRCT. The metastatic tumor displayed similar morphologic and immunophenotypic features to the retroperitoneal primary. H&E staining was performed on primary and metastatic tumors, and both displayed the classic DSRCT phenotype of small round blue cells surrounded by stromal desmoplasia (Figure 1(a)).

PCR primers were designed to amplify the *EWSR1-WT1* fusion gene to confirm the tumor identity as DSRCT. The *EWSR1* breakpoint location in DSRCT varies between intron 7 and intron 10, with intron 7 being the most commonly observed location [17]. The DSRCT breakpoint in *WT1* invariably occurs within intron 7 [17]. To account for all potential breakpoint locations, PCR primers were designed in exon 7 of *EWSR1* and exon 10 of *WT1* (Figure 1(b)). RNA was isolated from both the primary and recurrent patient tumors (denoted by 2911 and 2912, respectively) as well as from the DSRCT cell lines JN-DSRCT-1 and BER-DSRCT to serve as positive controls and a non-DSRCT undifferentiated round blue cell sarcoma (4JX1) to serve as a negative control. Reverse transcriptase (RT) was added or omitted during the cDNA synthesis of each tumor sample or cell line, and PCR was used to detect the presence of the *EWSR1-WT1* fusion gene. Specific DNA fragments were amplified in the JN-DSRCT-1 and BER-DSRCT positive controls as well as the tumor samples 2911 and 2912 but not in the negative control 4JX1 nor in the absence of RT (Figure 1(b)), thus confirming the tumor identity as DSRCT. The PCR amplicons for the primary and recurrent/metastatic tumor samples were identical and also the same size as the BER-DSRCT amplicon, suggesting the fusion in these tumors adjoined exon 7 of *EWSR1* to exon 8 of *WT1* as previously described for BER-DSRCT [13]. JN-DSRCT-1 generated a larger PCR product harboring the fusion between exon 10 of *EWSR1* and exon 8 of *WT1*, as described previously [13, 26]. Sanger sequencing of the PCR products further validated the precise joining of *EWSR1* exon 7 to *WT1* exon 8 in RNA transcripts from both the primary and metastatic tumor samples (Figure 1(c)).

### 3.2. EWSR1-WT1 Genomic Breakpoint Characterization

**Figure 2:**
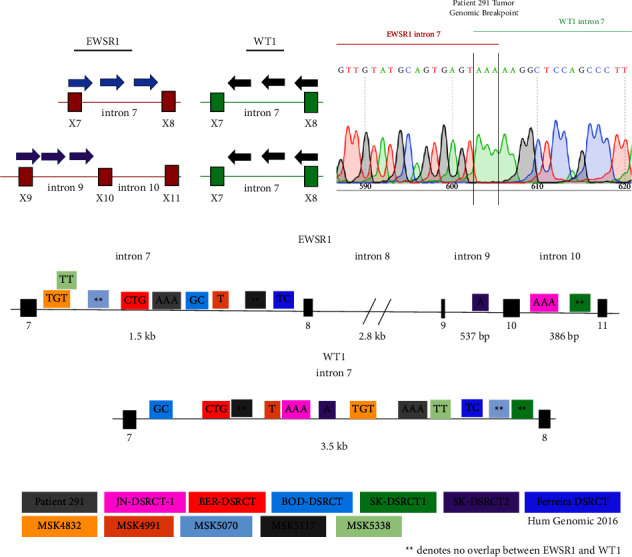
DSRCT genomic breakpoint map: (a) PCR strategy for amplifying the DSRCT genomic breakpoint using two different sets of *EWSR1* forward primers with the same common set of *WT1* reverse primers. (b) Histogram of Sanger sequencing results from the 291 patient's tumors showing a 3 bp overlap at the *EWSR1* intron 7 to *WT1* intron 7 genomic breakpoint junction. (c) DSRCT genomic breakpoint map showing the breakpoint location and base pair overlap for 5 DSRCT cell lines and 7 DSRCTs.

Having determined that the translocation in the patient tumors leads to an mRNA transcript that fuses exon 7 of *EWSR1* to exon 8 of *WT1*, we then sought to identify the exact genomic breakpoint location. We devised a strategy to identify the specific genomic breakpoints for the novel patient tumor, five additional DSRCTs and the five available DSRCT cell lines: JN-DSRCT-1, BER-DSRCT, BOD-DSRCT, SK-DSRCT1, and SK-DSRCT2. Given the sizes of *EWSR1* introns 7 and 8 (1.5 kb and 2.8 kb, respectively), it was impractical to design one set of PCR primers that can accurately amplify all genomic breakpoints of DSRCT. Therefore, our strategy utilized different primer sets depending on the location of the *EWSR1-WT1* fusion (Figure 2(a)). Different sets of forward primers were designed for intron 7 and intron 9 of *EWSR1*, and a common set of three reverse primers within the 3.5 kb region of intron 7 of *WT1* was used. The product of each successful PCR was then used for Sanger sequencing to identify the precise genomic breakpoints (Figure 2(b), Supplementary Figures 1 and 2).

The identified breakpoints were utilized to establish a map of known DSRCT genomic breakpoints which incorporates these 11 novel breakpoints as well as the one previously mapped breakpoint from the study of Ferreira et al. (Figure 2(c)) [18]. All identified breakpoints were unique with the exception of the *EWSR1* breakpoints of MSK4832 and MSK5338 which shared the same thymidine residue. The DSRCT genomic breakpoints were notable for the absence of insertions or deletions and the presence of only 1–3 base pairs of homology between the native *EWSR1* and *WT1* intron sequences that are joined. Four DSRCT genomic breakpoints had three shared base pairs at their junction (JN-DSRCT-1, BER-DSRCT, 291 DSRCT, and MSK4832), three DSRCT genomic breakpoints had two shared base pairs (BOD-DSRCT, Ferreira DSRCT, and MSK5338), and two DSRCT genomic breakpoint had one base pair overlap at its fusion breakpoint (SK-DSRCT2 and MSK4991). Three DSRCT samples had genomic breakpoints lacking microhomology (SK-DSRCT1, MSK5070, and MSK5117). Intriguingly, the SK-DSRCT1 junction contained “TA” of *EWSR1* intron 9 fused with “AT” of *WT1* intron 7 forming a 2-bp palindromic sequence. The lack of notable insertions or deletions and the presence of microhomologies at the breakpoints suggest a potential role for MMEJ in the formation of DSRCT translocations.

### 3.3. Recurrent DSRCT Upregulates RNA Splicing and Downregulates Immune System Response

**Figure 3:**
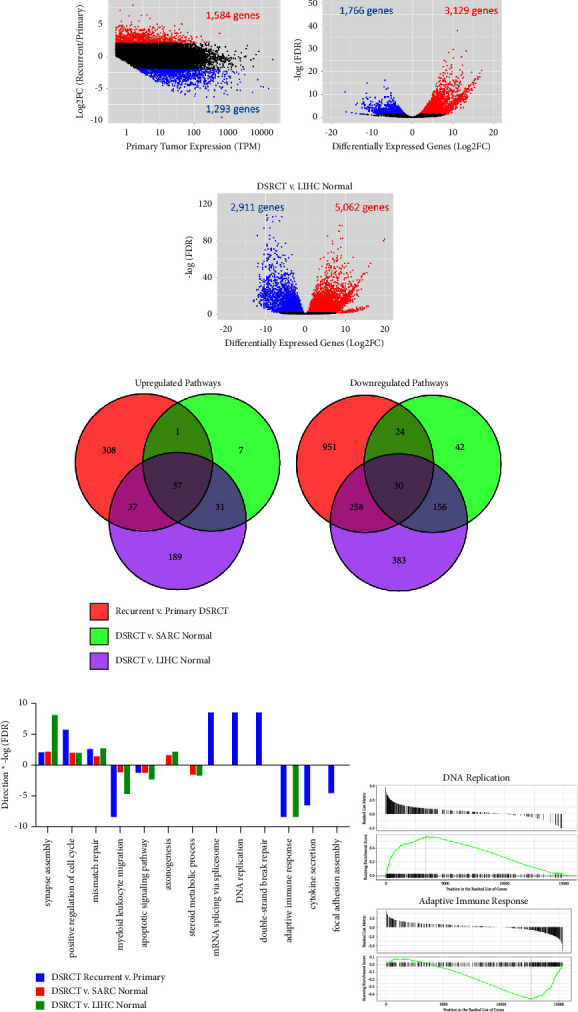
Primary versus recurrent DSRCT RNA sequencing analysis: (a) RNA sequencing analysis identified 1584 upregulated genes (>2 log2FC, red points) and 1293 downregulated genes (<−2 log2FC, blue points) in recurrent versus primary DSRCTs. Volcano plots of differentially expressed genes between DSRCT (*n* = 2) and (b) normal connective tissue (*n* = 2) or (c) normal liver (*n* = 5). (d) Venn diagrams comparing upregulated and downregulated GO-BP pathways as identified by GSEA on recurrent versus primary DSRCTs (red), DSRCT versus SARC normal tissues (green), or DSRCT versus LIHC normal tissues (purple). (e) Select significantly altered GO-BP pathways identified in (d) with GSEA. *Y*-axis indicates direction and magnitude of pathway enrichment where positive values indicate higher expression in recurrent versus primary DSRCT or in DSRCT versus normal tissues (plotted as direction ^*∗*^ -log(FDR). (f) GSEA on recurrent versus primary DSRCT showing positive enrichment of DNA replication and negative enrichment of adaptive immune response.

In addition to characterizing the breakpoint for the 291 DSRCT and using it in combination with established cell lines to gain insight into the formation of DSRCT, we also aimed to use this unique primary/recurrent model to understand pathways involved in DSRCT recurrence and metastasis. RNA sequencing was performed on the primary and metastatic tumor samples to unbiasedly examine alterations in gene expression. Genes in which both the primary and recurrent/metastatic tumor had expression >0.5 transcripts per million (TPM) were examined. 1,584 genes were identified as upregulated in the recurrent/metastatic tumor (>2 log2 foldchange), and 1,293 genes were identified as downregulated (<−2 log2foldchange) (Figure 3(a)). The gene expression of these tumor samples was also compared to two sets of normal tissues: normal connective tissue from The Cancer Genome Atlas Sarcoma dataset (SARC normal) and normal liver tissue from The Cancer Genome Atlas Liver Hepatocellular Carcinoma dataset (LIHC normal). Differential gene expression analysis identified over 3,000 genes more highly expressed in DSRCT compared to each of these normal tissues and over 1,500 genes expressed less prominently in DSRCT (Figures 3(b) and 3(c)). Many of the identified differentially expressed genes were in common between both normal tissue comparisons with 2,732 genes found to be commonly enriched and 1,051 genes commonly depleted in DSRCT compared to both SARC normal and LIHC normal (Supplementary Figure 3A)

Gene set enrichment analysis (GSEA) identified Gene Ontology Biological Processes (GO-BP) altered in DSRCT versus normal tissues and between recurrent and primary DSRCT (Figure 3(d), Supplementary Data 1). Thirty-seven pathways were found to be both enriched in DSRCT compared to normal tissues and upregulated in the recurrent tumor (Figure 3(d)). These commonly upregulated pathways include positive regulation of cell cycle, mismatch repair, and synapse assembly (Figures 3(d) and 3(e)). An additional 31 pathways, including axonogenesis and axon guidance, were enriched in DSRCT related to both the normal liver and normal connective tissue, but not upregulated in the recurrent tumor. We further identified 308 pathways that were uniquely enriched in recurrent versus primary DSRCT and not between DSRCT and normal tissues. A multitude of the pathways enriched in the recurrent tumor related to DNA replication, DNA repair, and RNA splicing. Enriched DNA replication-related pathways included DNA replication, DNA-dependent DNA replication, DNA recombination, and DNA packaging (Figures 3(e) and 3(f), Supplementary Data 1). Enriched DNA repair-related pathways included double-stranded break repair, homologous recombination, and nucleotide excision repair. Pathways related to RNA splicing were also highly enriched, with three pathways related to RNA splicing all having a *p* value <1*E* − 10. The highest ranking genes involved in altered RNA splicing were NOVA1, ELAVL2, SNRNP25, ESRP2, and SRRM4. GSEA on Kyoto Encyclopedia of Genes and Genome (KEGG) pathways revealed similar findings including upregulation of cell cycle, homologous recombination, and the spliceosome in recurrent versus primary DSRCT (Supplementary Figures 3B and 3C).

Conversely, 30 pathways were identified as reduced in DSRCT compared to the normal tissue and downregulated in the recurrent tumor, including myeloid leukocyte migration, apoptotic signaling, and cellular cation homeostasis (Figures 3(d) and 3(e)). The steroid metabolic process, carboxylic acid biosynthetic process, and various other metabolism-related pathways had reduced gene expression in DSRCT compared to the normal connective tissue and liver. Over 900 pathways were identified as uniquely downregulated in the recurrent DSRCT as compared to the primary tumor. Categories of pathways enriched in the primary versus recurrent tumor included immune system function, cell adhesion, and PI3-AKT signaling (Figure 3(e)). Notably, over 30 pathways related to both immune system function and cell interactions were identified as downregulated in recurrent versus primary DSRCT (Supplementary Data 1). Downregulated immune system-related pathways include TNF signaling pathway, macrophage activation, cytokine secretion, and regulation of T cell proliferation. Downregulated cell adhesion-related pathways include extracellular matrix organization, cell adhesion molecules, and focal adhesion. Pathways in these same categories were also identified with GSEA on KEGG pathways (Supplementary Figures 3B and 3C). The downregulation of the immune system function in the recurrent tumor may signal the ability of recurrent/metastatic DSRCT to avoid the immune system while downregulation of focal adhesion is consistent with a more invasive/metastatic phenotype.

### 3.4. The EWSR1-WT1 Signature Is Enriched in Recurrent, Metastatic DSRCT

**Figure 4:**
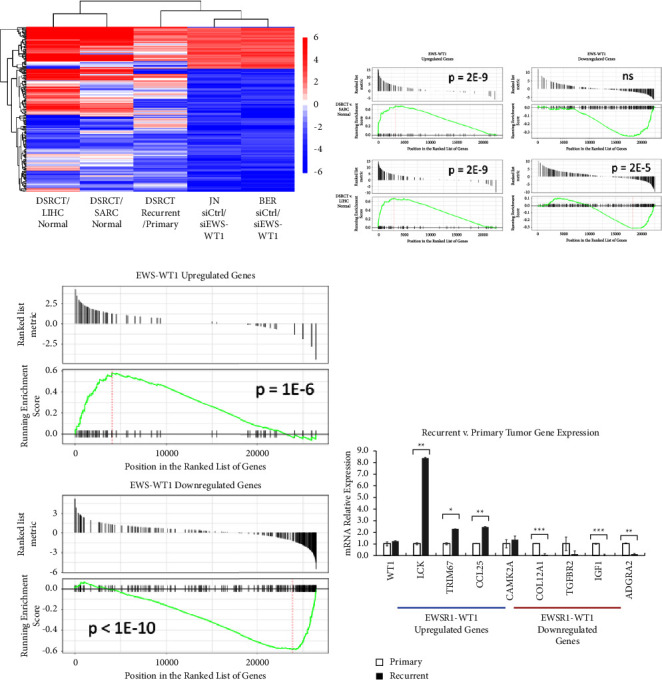
The EWSR1-WT1 signature in recurrent DSRCT: (a) heatmap of the *EWSR1-WT1*-regulated gene set identified by Gedminas et al. Log2FC of DSRCT versus LIHC normal tissues, DSRCT versus SARC normal tissues, recurrent versus primary DSRCTs, and siCtrl versus siEWSR1-WT1 in JN-DSRCT-1, and BER-DSRCT are plotted. (b) GSEA of DSRCT versus SARC normal tissues and DSRCT versus LIHC normal tissues on EWSR1-W*T1* upregulated and downregulated gene sets. (c) GSEA of recurrent versus primary DSRCT log2FC on *EWSR1-WT1* upregulated and downregulated gene sets. (d) RT-qPCR gene expression analysis of *EWSR1-WT1* (via *WT1* targeting primers) plus four *EWSR1-WT1* upregulated genes and four *EWSR1-WT1* downregulated genes in primary versus recurrent DSRCTs (*n* = 2, ^*∗*^*p* < 0.05, ^*∗∗*^*p* < 0.01, and ^*∗∗∗*^*p* < 0.001).

Recently, Gedminas et al. used siRNA knockdown of EWSR1-WT1 to identify downstream genes regulated by the fusion oncogene. Utilizing this gene set and the corresponding publicly available data, we compared the expression of EWSR1-WT1-regulated genes in DSRCT versus normal tissues and between DSRCT in the primary and recurrent/metastatic state. Concordant with the importance of the EWSR1-WT1 fusion in DSRCT transcriptional regulation, we found EWSR1-WT1 upregulated genes were highly expressed in DSRCT versus normal tissues and that EWSR1-WT1 downregulated genes were generally expressed at lower levels in DSRCT than normal tissues (Figures 4(a) and 4(b)). A heatmap of the EWSR1-WT1-regulated gene set demonstrates extremely strong enrichment of EWSR1-WT1 upregulated genes in DSRCT samples as compared to normal tissues, with many genes enriched >5 log2fold relative to the normal connective tissue and liver (Figure 4(a)). Remarkably, this enrichment in many cases had a larger magnitude than the change in expression observed in the Gedminas et al.'s dataset when the fusion protein was knocked down in JN-DSRCT-1 and BER-DSRCT cell lines. GSEA on the EWSR1-WT1 upregulated gene set confirmed this strong enrichment (*p* = 2*E* − 9). The negative enrichment of EWSR1-WT1 downregulated genes in DSRCT versus normal tissues was also observed but was less robust and more heterogeneous, with some of the genes normally downregulated by EWSR1-WT1 counterintuitively expressed more highly on DSRCT than on normal tissues. GSEA found statistically significant negative enrichment in DSRCT compared to the normal liver but not normal connective tissues (Figure 4(b)).

Intriguingly, the EWSR1-WT1 upregulated and downregulated gene sets were also identified as enriched when comparing the gene expression of primary versus recurrent/metastatic DSRCTs (Figure 4(a)). A large fraction of genes upregulated by EWSR1-WT1 was expressed at a higher degree in the recurrent/metastatic tumor versus the primary tumor. Similarly, many genes downregulated by EWSR1-WT1 were expressed at a lower level in the recurrent/metastatic tumor versus primary tumor. This observation was validated by GSEA of EWSR1-WT1 upregulated and downregulated gene sets, with *p* = 1*E* − 6 for genes upregulated by EWSR1-WT1 and *p* < 1*E* − 10 for genes downregulated by EWSR1-WT1 (Figure 4(c)). While this enrichment was statistically significant, not all EWSR1-WT1 targets demonstrated this behavior with some targets showing no alteration in gene expression between recurrent and primary tumors and a few demonstrating the opposite trend. RT-qPCR analysis validated these findings, demonstrating increased expression of genes that are normally upregulated by EWSR1-WT1 in the recurrent/metastatic tumors (*LCK*, *TRIM67*, *CCL25*, and *CAMK2A*), while finding reduced expression of genes normally downregulated by EWSR1-WT1 in the recurrent/metastatic tumors (*COL12A1*, *IGF1*, *TGFBR2*, and *ADGRA2*) (Figure 4(d)). Using qPCR primers in the C-terminus of *WT1*, we found a slight increase in the expression of *EWSR1-WT1* in the recurrent/metastatic tumor as compared to the primary tumor (∆∆Cq of 0.30) (Figure 4(d)). We note that endogenous *WT1* is not expressed in DSRCT cells (primary or cell lines), thus all WT1 expression in DSRCT can be attributed to EWSR1-WT1 [16]. Intriguingly, the increase in *EWSR1-WT1* target gene expression was to a greater magnitude than the increase in the *EWSR1-WT1* fusion gene itself, suggesting that mechanisms other than increasing fusion gene expression contribute to the observed upregulation of genes in the *EWSR1-WT1* gene set.

## 4. Discussion

DSRCT is a rare and extremely deadly pediatric cancer that remains understudied. Here, we report, to our knowledge, the first analysis comparing a primary DSRCT and its corresponding recurrent tumor at the molecular level. The recurrent tumor was located within the liver parenchyma and excised 5 years after primary tumor excision. Both the primary and recurrent tumors demonstrated a fusion between exon 7 of *EWSR1* and exon 8 of *WT1*, which previous studies have shown is the most common translocation for DSRCT [17]. We characterized the specific genomic breakpoint for this tumor pair, the five available DSRCT cell lines, and five other DSRCTs in order to establish the first DSRCT genomic breakpoint map. We further performed RNA sequencing and identified altered genes and pathways in the recurrent versus primary tumors that may shed light on the mechanisms of recurrence and metastasis in the DSRCT.

The DSRCT genomic breakpoint map provides several insights into the formation of this rare tumor. On chromosome 22, breakpoints were identified in EWSR1 introns 7, 9, and 10. This is consistent with previous publications identifying cases of DSRCT where either exon 7, 9, or 10 of EWSR1 is fused to exon 8 of WT1 [17]. It is notable that intron 8 of EWSR1 is substantially larger than introns 7, 9, and 10 (2.8 kb versus 1.5 kb, 0.5 kb, and 0.4 kb, respectively) and yet no genomic breakpoints were identified within this intron. If both DNA double-stranded break formation and subsequent translocation formation are random processes, it would be expected that translocations would occur more frequently in a genomic region that is larger in size. A likely explanation for this counterintuitive observation is that a fusion of *EWSR1* exon 8 to *WT1* exon 8 would produce a frameshifted transcript where the codons for the C-terminal domain of *WT1* are out of frame with the preceding codons for *EWSR1*. Thus, the resulting transcripts would fail to produce a functional *EWSR1-WT1* protein capable of binding to DNA and driving oncogenesis, unless an alternative splicing is utilized to exclude exon 8 of *EWSR1*.

Intriguingly, the genomic breakpoint map was remarkable in finding that 75% of DSRCT genomic breakpoints include a small (1 to 3 base pair) region of homology at the *EWSR1* to *WT1* junction. This is a higher rate of microhomology than that found in other fusion-based cancers including acute lymphocytic leukemia (24%), prostate cancer (46%), anaplastic large-cell lymphoma (38%), and Ewing sarcoma (46%) [20, 22, 33, 34]. DSRCT breakpoints were further notable for the complete absence of filler bases at their breakpoint junction. Filler bases have been identified in approximately 15–20% of breakpoints in other fusion-based tumors and are a byproduct of NHEJ [33, 35]. Together, these findings suggest DSRCT genomic breakpoints may form predominantly through MMEJ rather than NHEJ [36, 37].

NHEJ is typically the predominant double-stranded DNA repair mechanism in cells [35]. However, absence of critical members of the NHEJ machinery including Ku70, Ku80, and DNA Ligase 4 (LIG4) can prevent NHEJ and lead to double-stranded break repair via MMEJ [37, 38]. The finding of a high rate of microhomologies at DSRCT breakpoints could suggest that mutations or reduced expression in NHEJ machinery may predispose cells to *EWSR1-WT1* translocation formation and the development of DSRCT. Devecchi et al. performed whole exome sequencing on 6 DSRCTs and found an overrepresentation of mutations in pathways related to DNA damage response [39]. However, none of the altered genes they identified are members of the KEGG gene set for NHEJ [39]. Similarly, whole genome sequencing on 10 DSRCTs and MSK-IMPACT analysis on 68 tumors by Slotkin et al. as well as whole exome sequencing of 22 DSRCTs by Wu et al. failed to find mutations in NHEJ machinery [15, 17]. Chow et al. performed FoundationOne® Heme next generation sequencing on 83 DSRCTs and identified four DSRCT samples with copy number alterations in the NHEJ pathway member RAD50 but no mutations in other NHEJ members [40]. Together, these findings suggest that most DSRCTs do not have alterations in genes involved in NHEJ. A potential alternative explanation for the predominance of MMEJ in *EWSR1-WT1* translocation formation is that the DSRCT cell of origin, which to date remains unknown, has decreased expression of NHEJ machinery and/or increased expression of MMEJ machinery. MMEJ machineries including DNA ligase III (LIG3), DNA ligase I (LIG1), and poly(ADP-ribose polymerase 1) (PARP1) are highly expressed in neural crest stem cells, the cell of origin for neuroblastoma, and thought to contribute to the tumor's ability to survive double-stranded break triggering events [41, 42]. A role for MMEJ in DSRCT is also suggested by a study demonstrating that PARP1 is commonly expressed in DSRCT and that PARP1 inhibition with olaparib is an effective treatment in the DSRCT cell line JN-DSRCT-1, with an IC_50_ of 1.38 *μ*M [43]. Further research should examine the function of MMEJ in DSRCT both as a potential treatment target and to gain insights into the mechanism of DSRCT formation.

RNA sequencing of the primary and recurrent tumor pair identified a variety of pathway alterations that may provide the first mechanistic insights into DSRCT recurrence and metastasis. The top three Gene Ontology biological pathways all related to alterations in RNA splicing, an oncogenic mechanism that has been recently identified in a variety of solid tumors and hematologic malignancies [44]. Alternative splicing of CD44 under the control of splicing factor ESRP1 has been shown to increase migration and lead to metastasis in breast cancer, ovarian cancer, and melanoma [45–48]. Also, in breast cancer, SNRPA1 has been shown to alter the splicing of PLEC leading to enhanced lung colonization and cancer cell invasion [49]. However, to date, no study has examined alternative splicing in DSRCT. Our finding suggests alternative splicing as a potential mechanism behind DSRCT metastasis which warrants further investigation.

Two other trends noted in our pathway analysis were upregulation of pathways involved in DNA repair (recombinational repair, double-stranded break repair via homologous recombination, nucleotide excision repair, and mismatch repair) and downregulation of pathways related to the immune system (negative regulation of cytokine production, regulation of leukocyte migration, neutrophil mediated immunity, TNF signaling pathway, and allograft rejection). Upregulation of DNA repair pathways can help tumors resist chemotherapy and radiotherapy, leading to tumor recurrence [50]. Utilizing small molecules R1-1 and B02 to inhibit RAD51, which plays an important role in homologous recombination, has led to radiotherapy and chemotherapy sensitization in glioma and breast cancer, respectively. The addition of RAD51 inhibitors could be a valuable treatment option that could prevent upregulated DNA repair pathways from causing therapy resistance in DSRCT [50–52]. Checkpoint kinase 1 (CHK1) inhibitors also have the potential to overcome increased DNA repair activity by preventing the cell cycle arrest that allows DNA to be repaired before replication [50]. Recent studies have demonstrated the ability of CHK1 inhibitors to kill DSRCT cells both *in vitro* and *in vivo* and have led to an ongoing phase I clinical trial (NCT04095221) [53, 54]. Our finding of downregulation in immune-related pathways in recurrent/metastatic DSRCT is consistent with a recent study that found a low level of immune infiltration in DSRCT using ssGSEA and ESTIMATE scoring on DSRCT RNA-seq samples [15]. Our finding extends these results and suggests that not only is DSRCT immune-cold but that recurrent DSRCT has an even lower penetration of immune cells than primary tumors. Together, these findings suggest DSRCT, both in its primary and recurrent forms, is unlikely to be amenable to immune checkpoint inhibitors.

In addition to identifying a variety of altered pathways, our RNA-seq data enabled an examination of EWSR1-WT1 target genes. We found enrichment of the EWSR1-WT1 gene set in DSRCT compared to normal tissues as well as in recurrent versus primary DSRCT. Alterations in epigenetics and the microenvironment have been shown to influence gene expression of recurrent tumors in other cancer types and are valuable areas worthy of further exploration in DSRCT recurrence [55–57]. While a variety of adult cancers become less reliant on the initiating set of mutations as they progress and acquire new mutations, our observation that the EWSR1-WT1 signature is enhanced in recurrent/metastatic DSRCTs suggests recurrent tumors may actually be more reliant on the *EWSR1-WT1* fusion oncogene [58, 59]. EWSR1-WT1-regulated genes expressed more highly in recurrent than primary DSRCT included *GJB2*, *GAL*, and *GALP*, which were recently identified as highly enriched in DSRCT compared to other sarcoma types [60]. Consistent with our finding that the EWSR1-WT1 signature is enriched in recurrent DSRCT, we recently showed that DSRCT CSC-like cells, which may be involved in recurrent tumor formation, are dependent on the EWSR1-WT1 fusion protein [61]. Gedminas et al. demonstrated that the small molecule lurbinectedin inhibits the EWSR1-WT1 fusion *in vitro* by inducing nucleolar redistribution of the fusion protein [10]. In a case report, the related molecule trabectedin, when administered to patients in combination with irinotecan, led to complete remission in one patient and stable disease in another [62]. Our finding indicates that the fusion signature remains important in recurrent/metastatic tumors indicates that a EWSR1-WT1 targeting therapy such as lurbinectedin and trabectedin, or an antisense oligonucleotide may be effective in both primary and recurrent DSRCTs.

While our RNA-seq analysis identified a number of pathways that improve the understanding of DSRCT recurrence and suggest potentially useful therapies to prevent recurrence, our conclusions are limited by the small sample size of only one primary-recurrent tumor pair. The role of each of the identified pathways in DSRCT recurrence and metastasis should be validated on larger sets of primary-recurrent tumor pairs and further investigated to identify therapies that may prevent recurrence and metastasis, ultimately leading to improved clinical outcomes.

## 5. Conclusion

Lack of sample availability is a limiting factor in understanding DSRCT biology and designing effective therapies. Here, we present, to our knowledge, the first molecular characterization and transcriptomic analysis of a pair of primary and recurrent DSRCTs from the same patient. We identified the genomic breakpoint of this novel tumor pair, five DSRCT cell lines and five additional tumors. The high rate of 1–3 bp microhomologies at DSRCT genomic breakpoints suggests a role of MMEJ in fusion gene formation, providing insights into the potential DSRCT cell of origin. Through RNA-sequencing analysis, we conducted the first comparison of gene expression alterations between recurrent and primary DSRCT and identified pathway alterations that may help explain DSRCT recurrence, including upregulation of DNA damage repair and mRNA splicing as well as downregulation of immune system function and focal adhesion. Our analysis of genes regulated by the *EWSR1-WT1* fusion protein suggests the fusion protein remains the principal driver in DSRCT recurrence with enrichment in genes regulated by the fusion protein in the recurrent versus primary tumor.

## Data Availability

The RNA-seq dataset generated and analyzed during the current study is available on GEO at GSE230603.

## Supplementary Materials

Supplementary Materials Supplementary Figure 1: DSRCT cell line genomic breakpoints. Histograms of Sanger sequencing results from DSRCT cell lines: JN-DSRCT-1, BER-DSRCT, BOD-DSRCT, SK-DSRCT1, and SK-DSRCT2. Red and green lines show the original sequence of EWSR1 and WT1 introns, respectively. Region between the black vertical lines shows the microhomology shared by both EWSR1 and WT1 introns, if any. Supplementary Figure 2: DSRCT genomic breakpoints. Histograms of Sanger sequencing results from DSRCTs: MSK4832, MSK4991, MSK5070, MSK5338, and MSK5117. Red and green lines show the original sequence of EWSR1 and WT1 introns, respectively. Region between the black vertical lines shows the microhomology shared by both EWSR1 and WT1 introns, if any. Supplementary Figure 3: DSRCT genomic breakpoints. (A) Venn diagrams identifying commonly upregulated and downregulated genes between DSRCT versus SARC normal tissues(green) and DSRCT versus LIHC normal tissues(purple). Genes were considered differentially expressed if *p* < 0.05 and |log2FC| > 2. (B) Top 15 KEGG-enriched pathways between recurrent and primary DSRCTs from GSEA. (C) GSEA of KEGG pathways on recurrent versus primary DSRCTs showing upregulation of cell cycle, homologous recombination, and the spliceosome, while showing downregulation of TNF signaling, cytokine receptor interaction, and focal adhesion. Supplementary Table 1: DSRCT patient samples. Supplementary Table 2: PCR primers. Supplementary Table 3: RT-qPCR primers. Supplementary Data 1: DSRCT pathway enrichment analysis.
