# Supplementary material for: Genomic Breakpoint Characterization and Transcriptome Analysis of Metastatic, Recurrent Desmoplastic Small Round Cell Tumor
Source: Sarcoma. 2023 Jul 6;2023:6686702. doi: 10.1155/2023/6686702 (PMC10344636; doi:10.1155/2023/6686702)
Supplement: Supplementary Materials — Supplementary Figure 1: DSRCT cell line genomic breakpoints. Histograms of Sanger sequencing results from DSRCT cell lines: JN-DSRCT-1, BER-DSRCT, BOD-DSRCT, SK-DSRCT1, and SK-DSRCT2. Red and green lines show the original sequence of EWSR1 and WT1 introns, respectively. Region between the black vertical lines shows the microhomology shared by both EWSR1 and WT1 introns, if any. Supplementary Figure 2: DSRCT genomic breakpoints. Histograms of Sanger sequencing results from DSRCTs: MSK4832, MSK4991, MSK5070, MSK5338, and MSK5117. Red and green lines show the original sequence of EWSR1 and WT1 introns, respectively. Region between the black vertical lines shows the microhomology shared by both EWSR1 and WT1 introns, if any. Supplementary Figure 3: DSRCT genomic breakpoints. (A) Venn diagrams identifying commonly upregulated and downregulated genes between DSRCT versus SARC normal tissues(green) and DSRCT versus LIHC normal tissues(purple). Genes were considered differentially expressed if p < 0.05 and |log2FC| > 2. (B) Top 15 KEGG-enriched pathways between recurrent and primary DSRCTs from GSEA. (C) GSEA of KEGG pathways on recurrent versus primary DSRCTs showing upregulation of cell cycle, homologous recombination, and the spliceosome, while showing downregulation of TNF signaling, cytokine receptor interaction, and focal adhesion. Supplementary Table 1: DSRCT patient samples. Supplementary Table 2: PCR primers. Supplementary Table 3: RT-qPCR primers. Supplementary Data 1: DSRCT pathway enrichment analysis. [file 6686702.f1.zip › Supplementary Figures and Tables Revised.docx]

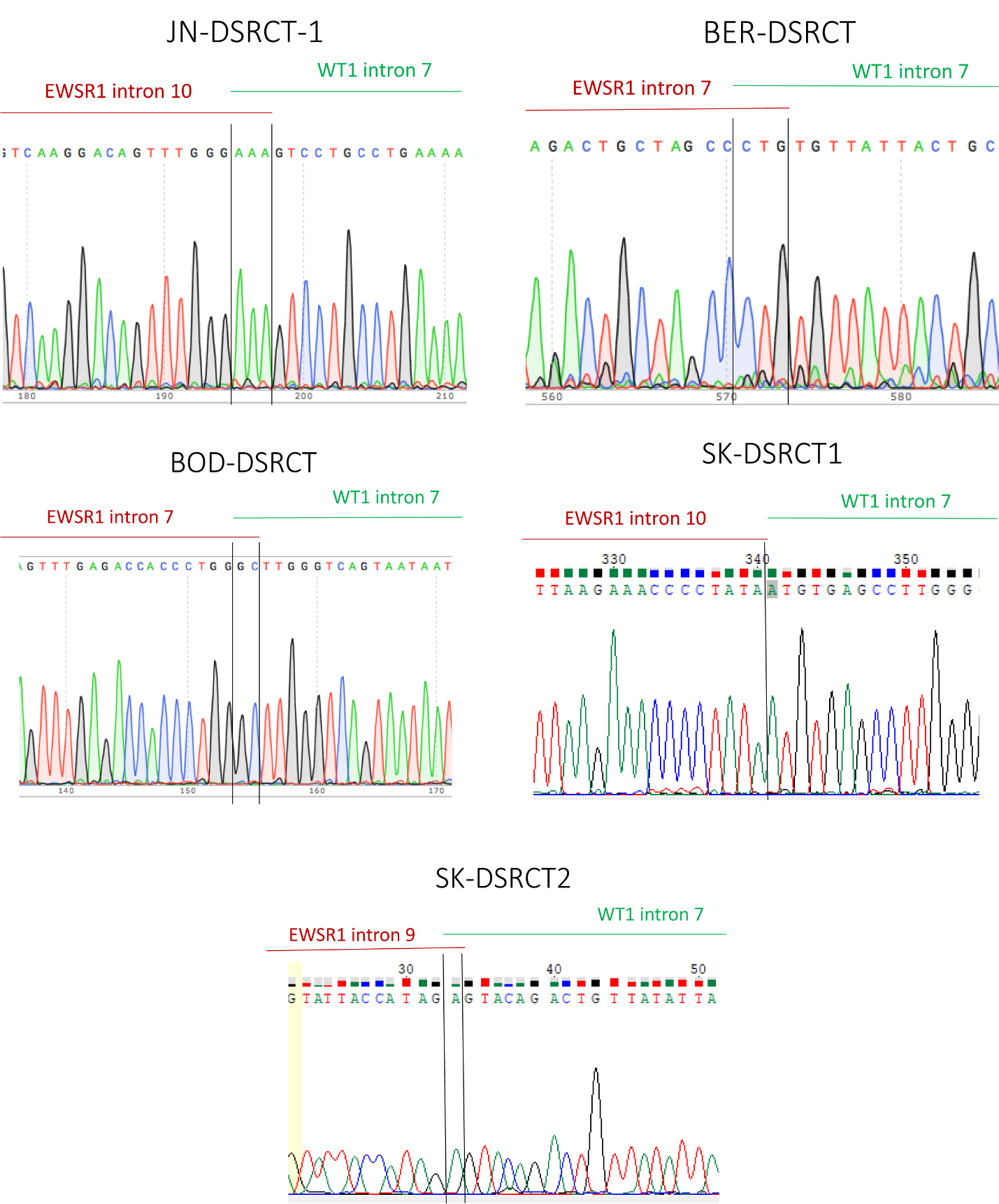


**Supplementary Fig. 1. DSRCT Cell Line Genomic Breakpoints**. Histograms of Sanger sequencing results from DSRCT cell lines JN-DSRCT-1, BER-DSRCT, BOD-DSRCT, SK-DSRCT1, and SK-DSRCT2. Red and green lines show the original sequence of *EWSR1* and *WT1* introns, respectively. Region between the black vertical lines shows the microhomology shared by both *EWSR1* and *WT1* introns, if any.


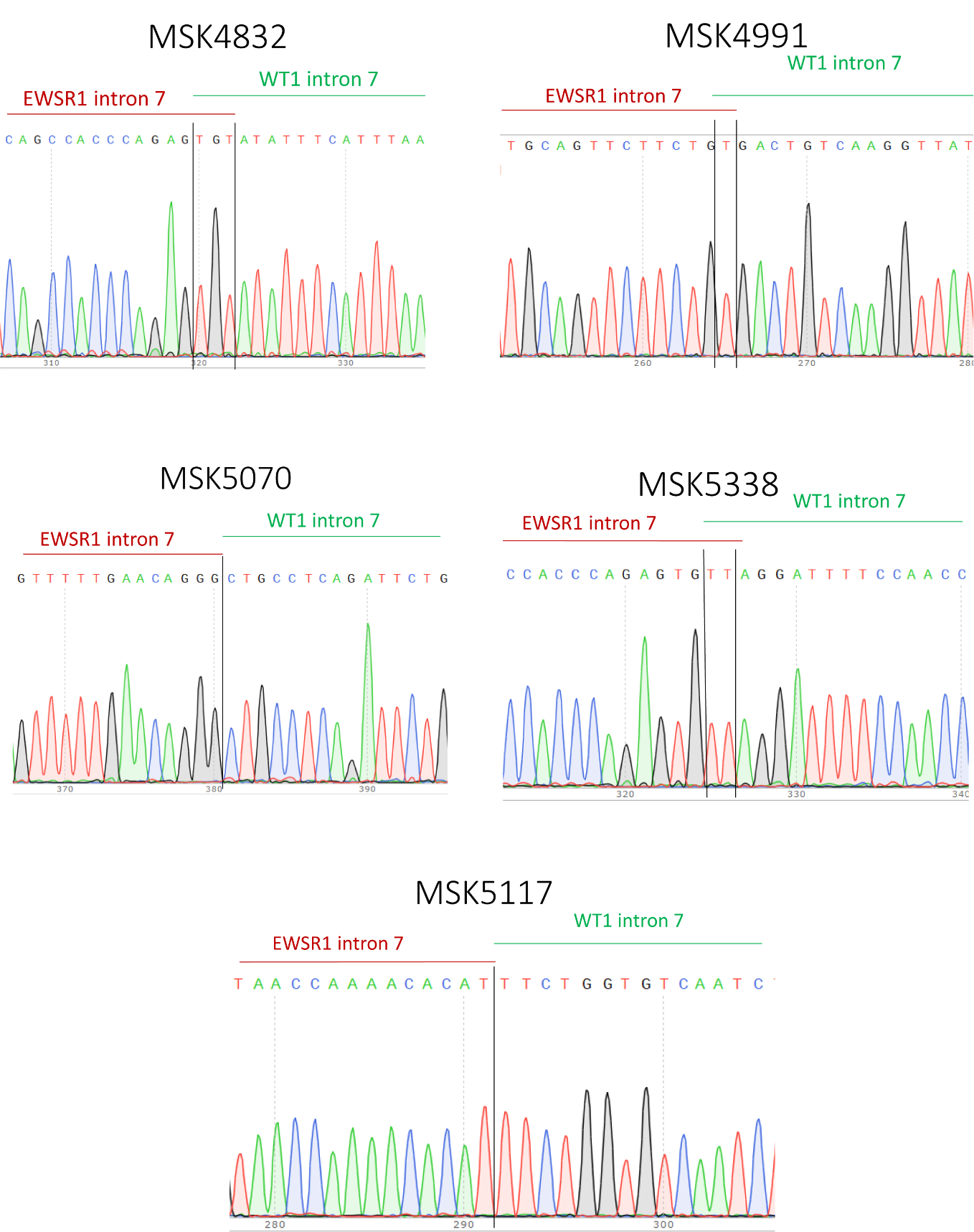


**Supplementary Fig. 2. DSRCT Tumor Genomic Breakpoints**. Histograms of Sanger sequencing results from DSRCT tumors: MSK4832, MSK4991, MSK5070, MSK5338, and MSK5117. Red and green lines show the original sequence of *EWSR1* and *WT1* introns, respectively. Region between the black vertical lines shows the microhomology shared by both *EWSR1* and *WT1* introns, if any.


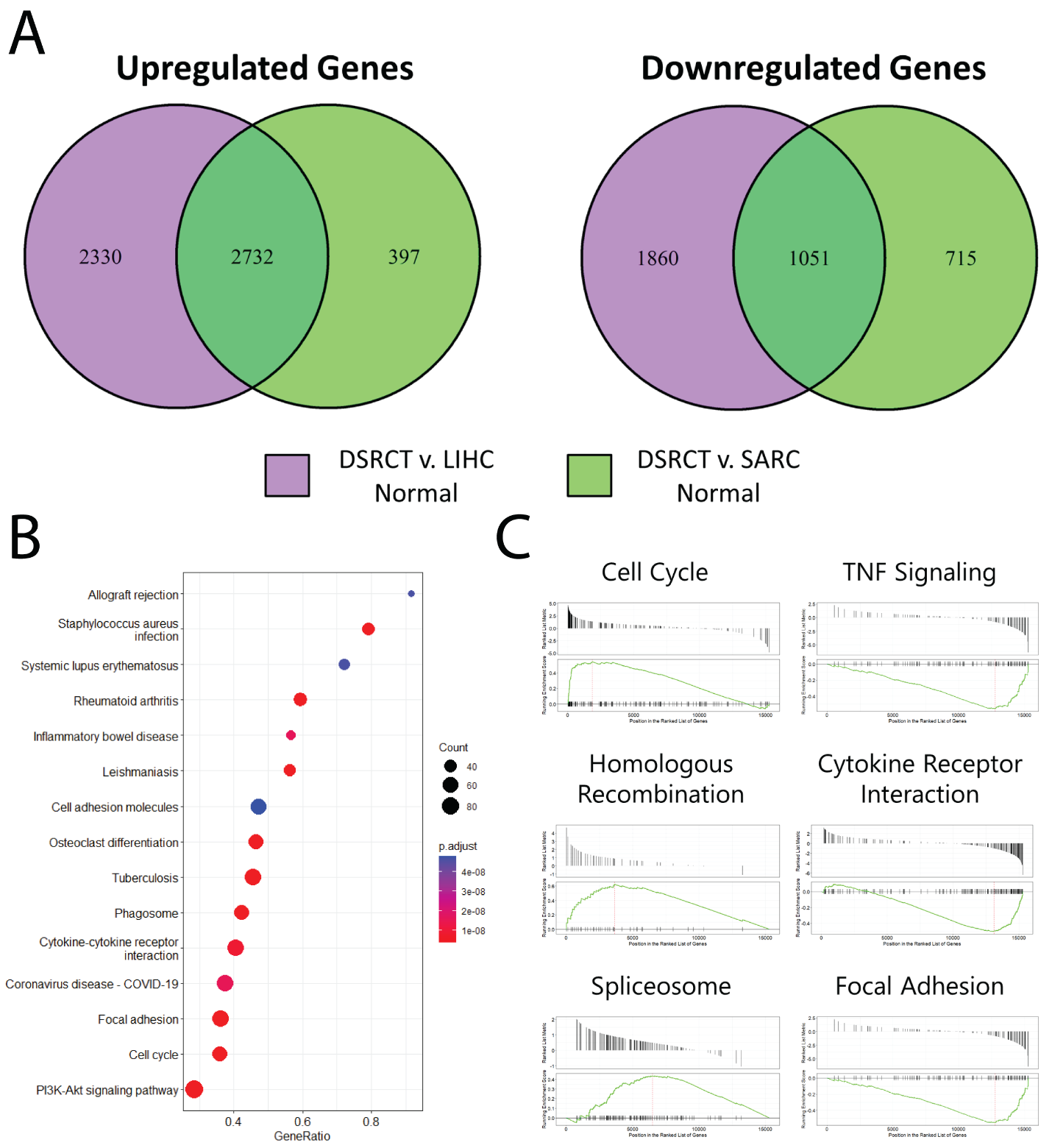


**Supplementary Fig. 3. DSRCT Tumor Genomic Breakpoints**. **(A)** Venn diagrams identifying commonly upregulated and downregulated genes between DSRCT v. SARC Normal (green) and DSRCT v. LIHC Normal (purple). Genes were considered differentially expressed if p<0.05 and |log2FC| > 2. **(B)** Top 15 KEGG enriched pathways between recurrent and primary DSRCT tumors from GSEA. **(C)** GSEA of KEGG pathways on recurrent versus primary DSRCT tumors showing upregulation of cell cycle, homologous recombination, and the spliceosome, while showing downregulation of TNF signaling, cytokine receptor interaction, and focal adhesion.

**Supplementary Table 1:** DSRCT Patient Tumor Samples

| **Patient** | **Tumor Location** | **Age at Tumor Harvest** | **Sex** |
| --- | --- | --- | --- |
| MSK4832 | Pelvic cavity | 8 | Male |
| MSK4991 | Mesenteric implant | 23 | Male |
| MSK5070 | Omentum | Unknown | Male |
| MSK5117 | Omentum | 30 | Male |
| MSK5338 | Pelvic cavity | 12 | Male |

**Supplementary Table 2:** PCR Primers

| **Primer Name** | **Primer Sequence (5’ -> 3’)** |
| --- | --- |
| hEWSR1-x7i7-F | AGCTACGGGCAGCAGAGTGAGTTGC |
| hEWSR1-i7-665-F | GAGGCAGCTATTGCAGGCCACTATG |
| hEWSR1-i7-1090-F | CCCCTAGCAGTGTGCTAAAGCCC |
| hEWSR1-i8x9-F | AGCAGCGCTGGAGAGCGAGG |
| hEWSR1-x9i9-F | GCTTCAATAAGCCTGGTGGTAAG |
| hEWSR1-i9-125-F | GTACTGCCGGCATTGTCTTAGG |
| hEWSR1-i9-473-F | GTAAGGTTTGTAGCTTGCAAGACGTGCAC |
| hWT1-i7-864-R | CCAGAAGCAGCCTCTCTGACCTG |
| hWT1-i7-2391-R | GGAAAATCCTAAGGGCTGGAGCC |
| hWT1-i7-3443-R | GGCAACCTCTCCTACTAGGACTGAACAG |

**Supplementary Table 3:** RT-qPCR Primers

| **Gene** | **Fwd Primer (5’ -> 3’)** | **Rev Primer (5’ -> 3’)** |
| --- | --- | --- |
| ACTB | GCAAAGACCTGTACGCCAAC | AGTACTTGCGCTCAGGAGGA |
| WT1 (c-term)/ EWSR1-WT1 | CCATACCAGTGTGACTTCAAGG | TGTGGGTCTTCAGGTGGTC |
| LCK | GCTGACGGAAATTGTCACCC | TCAAGGCTGAGGCTGGTACT |
| TRIM67 | CAGGAGCAGTGGGTCAAAGG | CAGTAGGGGGACGGGTGG |
| CCL25 | CTTGACCCAGTGGATATCGGT | GAGCACAGCCCACCCAAT |
| CAMK2A | ATGACAGCCTTCGAACCTGAG | GTGGGGATTCAGGATGGTGG |
| COL12A1 | CCAGGGTCCACAGGTTCAAG | AAGCAGCACTGGCGACTTAG |
| TGFBR2 | CTCGGTCTATGACGAGCAGC | CTGGGCCTCCATTTCCACAT |
| IGF1 | ACATTGCTCTCAACATCTCCCA | GGAGGACATGGTGTGCATCT |
| ADGRA2 | GCAATAACAAGATCACGGGGC | GCTGCACTGTGCTGATGATG |
